# Supplementary material for: Nexus between constructs of social cognitive theory model and diabetes self-management among Ghanaian diabetic patients: A mediation modelling approach
Source: PLOS Glob Public Health. 2022 Jul 25;2(7):e0000736. doi: 10.1371/journal.pgph.0000736 (PMC10022127; doi:10.1371/journal.pgph.0000736)
Supplement: S1 Questionnaire — (DOCX) [file pgph.0000736.s002.docx]

**QUESTIONNAIRE**

**Association between constructs of social cognitive theory model and diabetes self-management among Ghanaian diabetic patients**

Good morning/afternoon and thank you for your permission. We are final year nursing students of Garden City University College, Kenyasi-Ghana. We are conducting a research on the topic ‘**Association between constructs of social cognitive theory model and diabetes self-management among Ghanaian diabetic patients’** under the department of Nursing in partial fulfilment of our being awarded a BSc. Nursing Degree. We would be grateful if you could help by answering this questionnaire. Your contribution is valuable. We assure you that all information given by you will be strictly confidential and used for the purpose of this study alone. Thank you.

**Tick the appropriate response in the questions below.**

**SECTION A: Socio-demographics characteristics**

1. Age: ..........
2. Gender: Male [ ] Female [ ]
3. What is your occupation? Farming
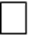
 Teaching
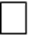
 Trading
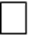
 Pensioner
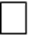
 Unemployed
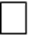
 others
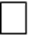

4. What is your marital status?

Married
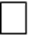
 Single
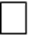
 Divorced
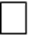
 Widow/Widower
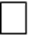


1. What is your religious status? Christian
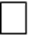
 Muslim
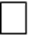
 Traditional
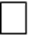

2. What is your highest level of education?

Primary school
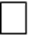
 JHS
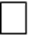
 SHS
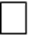
 Training college
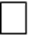
 University or higher
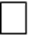


Others
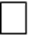
 Specify……………..

1. Do you have regular source of income? Yes
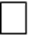
 No
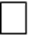

2. If yes, do you earn low (<500GHS) [ ], or Middle (500-999 GHS) [ ] or High (≥1000 GHS) [ ]
3. Who sponsors your hospital needs? NHIS
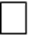
 Relatives
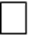
 Self
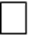
 Others
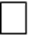
 specify...............................................
4. How do you feel your health is? Excellent [ ] Very good [ ] Good [ ] Fair [ ] Poor [ ]

**SECTION B: Health Profile**

1. Duration of having diabetes mellitus? ……………………….
2. Do you have any other chronic illness? Yes [ ] No [ ]
3. If yes what are they ……………………………….
4. Do you have any diabetes-related complications Yes [ ] No [ ]

If yes what are they ……………………………………

1. Latest HbA1c………………………………………………………………..
2. Current fasting blood sugar……………………………………………………
3. Type of Treatment……………………………………………………………..

**Knowledge of Diabetes**

1. Normal blood sugar is between 90-130mg/dL Yes [ ] No [ ] Don’t Know [ ]
2. If you feel thirsty, tired and weak, it usually means your blood sugar is high

Yes [ ] No [ ] Don’t Know [ ]

1. The best time to take insulin or diabetes pills is 15-30 min before a meal

Yes [ ] No [ ] Don’t Know [ ]

1. Insulin and diabetes pills make blood sugar go down Yes [ ] No [ ] Don’t Know [ ]
2. A person with diabetes check their feet for blisters or sore spot every day

Yes [ ] No [ ] Don’t know [ ]

1. When you exercise, your blood blood sugar goes down

Yes [ ] No [ ] Don’t know [ ]

1. If you feel shaky, sweaty and hungry, it usually means your blood sugar is low

Yes [ ] No [ ] Don’t know

1. If you suddenly get sweaty, nervous and shaky, you should eat some form of sugar

Yes [ ] No [ ] Don’t know [ ]

1. If diabetes is not well controlled, it can injure both kidneys and nerves

Yes [ ] No [ ] Don’t Know [ ]

1. You should get your eyes checked every year Yes [ ] No [ ] Don’t know [ ]
2. If you wake up in the morning, and you feel sick to your stomach and do not want to eat,, you should take half of the usual dose of medicine Yes [ ] No [ ] Don’t know [ ]

**Section C: Assessement of Diabetes Self Management (DSM)**

| On how many of the last seven days | 0 | 1 | 2 | 3 | 4 | 5 | 6 | 7 |
| --- | --- | --- | --- | --- | --- | --- | --- | --- |
| Did you follow the prescription of oral hypoglycaemic |  |  |  |  |  |  |  |  |
| Did you follow the prescription of insulin injections |  |  |  |  |  |  |  |  |
| Have you followed a diabetic diet |  |  |  |  |  |  |  |  |
| Have you eaten your meals at the same time each day |  |  |  |  |  |  |  |  |
| Did you participate in at least 30 minutes of exercise |  |  |  |  |  |  |  |  |
| Did you participate in a specific exercise session other than you do around the house or as part of your work |  |  |  |  |  |  |  |  |
| Did you test your blood urine sugar |  |  |  |  |  |  |  |  |
| Did you test your blood urine sugar the number of times recommended by your doctor |  |  |  |  |  |  |  |  |
| Did you check your feet |  |  |  |  |  |  |  |  |
| Did you dry between your toes after washing |  |  |  |  |  |  |  |  |

**Section D: Beliefs in Treatment Effectiveness**

|  | Not Important | Slightly Important | Fairly Important | Very Important | Extreme Important |
| --- | --- | --- | --- | --- | --- |
| How important do you believe that diabetic diet is for controlling blood glucose level |  |  |  |  |  |
| How important do you believe exercise is for controlling blood glucose levels |  |  |  |  |  |
| How important do you believe taking oral medications or injecting insulin is for controlling blood dlucose level |  |  |  |  |  |
| How important do you believe self-monitoring blood or urine glucose is for controlling blood glucose level |  |  |  |  |  |
| How important do you believe ecxercise is for preventing diabetic complications |  |  |  |  |  |
| How important do you believe taking medications or injecting insulin is for preventing diabetic complications |  |  |  |  |  |
| How important do you believe self-monitoring blood or urine glucose is for diabetic complications |  |  |  |  |  |
| How important do you believe checking your foot is for preventing diabetic complications |  |  |  |  |  |

**SECTION E:Respondent level of self-efficacy**

|  | Yes Defintely | Probably Yes | Maybe Yes Maybe No | Probably No | Definitely Not |
| --- | --- | --- | --- | --- | --- |
| I think I am able to check my blood urine glucose |  |  |  |  |  |
| I think I am able to follow my diabetic diet most of the time |  |  |  |  |  |
| I think I am able to follow my diabetic diet when I dine out together with my friends |  |  |  |  |  |
| I think I am able to examine my feet lesion |  |  |  |  |  |
| I think I am able to sufficient physical activities |  |  |  |  |  |
| I think I am able to take extra exercise when the doctor advises me to do so |  |  |  |  |  |
| I think I am able to take my medicine or inkect the insulin as prescribed |  |  |  |  |  |

SECTION F: Perceived of family support

|  | Never | Rarely | Sometimes | Often | Always |
| --- | --- | --- | --- | --- | --- |
| **How often did** your family listen carefully to what you have to say about diabetes |  |  |  |  |  |
| **How often did** your family buy or cook for you that especially recommended for your diabetes |  |  |  |  |  |
| **How often did** your family encourage you to participate in exercise |  |  |  |  |  |
| **How often did** your family select food choices required by diabetic diet when you ate with them |  |  |  |  |  |
| **How often did** your family praise you for sticking to following diabetic diet, exercising and self-monitoring blood/urine glucose |  |  |  |  |  |
| **How often did** your family help you remember to take your oral medicine or inject insulin |  |  |  |  |  |
| **How often did** your family help you to check your feet |  |  |  |  |  |

**SECTION G:Healthcare provider-patient communications**

| Over the past 3 months | Never | Rarely | Sometimes | Often | Always |
| --- | --- | --- | --- | --- | --- |
| **How often did** your healthcare provider use medical words that you did not undestand |  |  |  |  |  |
| **How often did** you have trouble of understanding your healthcare proviser because he she spoke too fast |  |  |  |  |  |
| **How ofen did** your healthcare provider listen to what you had to say about diabetes |  |  |  |  |  |
| **How often did** your healthcare provider answer your questions and address your concern about diabetes management during your visits |  |  |  |  |  |
| **How often** did your healthcare provider thoroughly explain why a test was being done and what were the results of the test your had done (eg HbA1c) |  |  |  |  |  |
| **How often did** your healthcare provider explain what you need to do take care of your diabete |  |  |  |  |  |
| How often fif your healthcare provider explain to you how to take medicine (oral agents or insulin) (when, how, and for how long |  |  |  |  |  |
