# Supplementary material for: Nexus between constructs of social cognitive theory model and diabetes self-management among Ghanaian diabetic patients: A mediation modelling approach
Source: PLOS Glob Public Health. 2022 Jul 25;2(7):e0000736. doi: 10.1371/journal.pgph.0000736 (PMC10022127; doi:10.1371/journal.pgph.0000736)
Supplement: Attachment — Submitted filename: Response to Reviewers.docx [file pgph.0000736.s003.docx]

**Response to Editorial comments**

**Dear Editor-In-Chief**

Manuscript tittle: **Nexus between constructs of social cognitive theory model and diabetes self-management among Ghanaian diabetic patients: A mediation modelling approach.**

**Article # PGPH-D-21-00139**

**We sincerely thank the reviewers for the truly helpful comments to improve the quality of our manuscript. We have read through carefully, please find our detailed responses below each specific point**

Reviewer # 1

This manuscript identifies factors influencing DSM practices among Type 2 diabetes mellitus (T2DM) patients in Ghana using social cognitive theory (SCT) in a hospital-based cross-sectional study. Most participants (65.5%) had high (>6.1mmol/L) fasting blood glucose (FBG) with an average of 6.93(SD=2.41). The path analysis model revealed that age ( p = 0.176 ), gender ( p = 0.901 ), and duration of T2DM ( p = 0.119 ) did not confound the relationships between the SCT constructs and DSM specified in the model. A significant direct positive effect of family and friends’support (Critical ratio (CR) = 5.279 , p < 0.001 ) on DSM was observed. Self-efficacy was a significant mediator in this relationship ( CR = 4.833, p < 0.001 ). There were significant conditional indirect effects (CIE) for knowledge of T2DM and family and friends’ support at medium and high levels of belief in treatment effectiveness (p < 0.05) via level of self-efficacy on DSM practices. However, no evidence of moderated mediation was observed for the exogenous variables on DSM.

**Comment**: Although this article appears to relevant in the field there are serious issues in the background, methods, and the discussions that need to be addressed. There are few grammatical errors too. Details are found below

**AUTHORS RESPONSE:** **The authors appreciate your comments. Prevalence rate has been corrected as prevalence.**

Details:

The title of the paper needs to be revised. I do not think the paper is looking at the relationship between health promotion constructs and diabetes self-management. Rather, they are using the SCT model to identify predictors of DSM. Please, revise your title.

**AUTHORS RESPONSE:** **The authors appreciate your comments. The title has been modified**

**Comment:** The background appears too long and a bit non-focused. The authors also provided 2 different conceptual frameworks with no narrative except for definitions of variables. The authors should revise the background by clearly articulating the problem. Do educate readers on the current strategies in Ghana to curb the diseases, and discuss whether these measures have been effective or not. If these programs have not been successful, explain why. Also state previous models that have been used to study this subject and further explain why the use of the SCT model to study predictors of your outcome is better. You also stated that findings from previous studies are inconsistent. Do explain why this is so. The authors also provided 2 conceptual frameworks. Use 1 (fig 2), and provide a clear and concise explanation of your conceptual framework; carefully aligning it to the background and your objectives/hypotheses.

(Your conceptual framework should explain the complex relationship between your dependent and independent variables. What I am seeing here is rather definitions of your variables (dependent and independent variables) which is fit for the methods section).

**AUTHORS RESPONSE:** **The authors appreciate your comments. The background has section has been revised based on the reviewer’s comments**

**Comment:** Also, note that the denominator of prevalence is not time. Change prevalence rate to simply prevalence.

**AUTHORS RESPONSE:** **The authors appreciate your comments. Prevalence rate has been changed to prevalence**

Methods

**Study Design/Area**

The study is not descriptive. Probably, a hospital-based cross-sectional design. Educate the reader on the following: How many Diabetes clinics are there in the Ashanti region? What proportion utilized the KATH Clinic? The KATH Clinic serves which part of the region, and so on. On average how many patients visit the clinic a year? These issues are linked to important selection bias.

**AUTHORS RESPONSE:** **The authors are grateful for your comments. The section has been revised as,** ’ **The study was a hospital-based cross-sectional study design undertaken from November 2018 to April 2019 at the Diabetes Clinic of Komfo Anokye Teaching Hospital (KATH) in the Ashanti Region of Ghana, Kumasi. KATH is located in Kumasi, the Regional Capital of Ashanti Region with a total projected population of 4,780,380 (2010 Ghana Population Census). KATH is the second-largest hospital in Ghana and Diabetes Clinic is part of the medical directorate that provides services to about 250 patients from Monday to Friday per week with an average of 10,706 out-patient attendance per year. The geographical location of the thousand two hundred- (1200-) bed capacity, the road network of the country, and commercial nature of Kumasi make the hospital accessible to all the areas that share the boundaries with Ashanti Region and others that are further away. KATH takes direct referrals from 12 out of the 16 administrative regions in Ghana. These are the Ashanti, Bono, Bono East, Ahafo, Western North, Savannah, Northern, North East, Upper East, Upper West and some parts of the Central and Eastern regions of Ghana. It also receives patients from neighbouring countries such as Ivory Coast and Eastern FasoThe diabetic centre of the KATH is situated beneath the medicine block (D block) just between the chest clinic and diagnostic centre and behind the emergency unit of the hospital”**

**Study Population**

How was your population sampled? On average how many patients visit the clinic a year? How were physically and mentally challenged patients determined?

**AUTHORS RESPONSE:** **The authors appreciate your comments. The section has been revised as**, ’ **A simple randomised sampling was used to recruit a total of 420 T2DM participants aged 30 years and above who visited the KATH Diabetic Clinic for routine check-ups and treatment. This study was conducted in consultation with clinicians and qualified health professionals. T2DM was diagnosed by clinicians at KATH and it was established based on the international classification of disease (ICD-10-CM Diagnosis Code E11.9). Each patient was carefully examined and their medical records thoroughly reviewed. As a result, we excluded all those individuals who were suffering from cancer, arthritis, infectious diseases, cardiovascular disease, thyroid disorders, pituitary disorders and adrenal disorders. The study did not include pregnant and lactating mothers. Since T2DM is largely a disease of ageing, the study recruited only individuals who were 30 years and above. Furthermore, to limit potential confounding and the likelihood of recruiting participants with type 1 diabetes,we excluded participants on insulin injections. Patients who were physically or mentally challenged and those who had less than 6 months duration of diabetes were excluded.**

Have a subsection for sampling strategy where you explain in detail how your population was sampled. Just stating terminology is not sufficient.

**AUTHORS RESPONSE:** **The authors appreciate your comments. This section has been included and given a new heading, ‘Study Population and subject sampling strategy’**

**Sample size justification**

Why did not you use the adherence rate? I guess the authors meant the proportion of diabetes patients practicing self-management? No data on this in Ghana? If the data is not available state that here and explain why you took data from an Ethiopian population. The reason for increasing your sample size from 366 to 420 should be stated clearly. I don't think statistical power and random error are the reasons.

**AUTHORS RESPONSE:** **The authors appreciate your comments. Regarding the reason for the sample size, believe that sample size increases with increasing power. Also, in statistics, there are two important ideas regarding sample size and margin of error. First, sample size and margin of error have an inverse relationship. Second, after a point, increasing the sample size beyond what you already have gives you a diminished return, because the increased accuracy will be negligible.**

Your data collection lacks a lot of details. Where was the interview carry? From what time to what time? Who carried out the interview and whether the person was provided training. How the interview session took place and some details about the different categories of questions, and so on.

Who validated the instrument: provide some details of this.

**AUTHORS RESPONSE:** **The authors appreciate your comments. The sections have been revised as, A self-reported questionnaire and validated questionnaires were used for the data collection on study variables. The first part of the questionnaire consisted of 28 questions divided into three sections including section A: demographic characteristics, section B: health profile, and section C: knowledge of T2DM. The second part consisted of 39 validated questions that measured DSM practices and SCT constructs. The DSM practices and SCT questionnaires have been validated, respectively. In addition, we assessed the reliability of the questionnaires in our setting as described in the sections below. The questionnaire was completed by each patient via a face-to-face interview approach by ethically recognised researchers and in consultation with clinicians. All interviews and data were collected at the Diabetic Clinic of the KATH. Interviews were conducted during the morning hours for the regular diabetic Clinic at the KATH.**

I see this sampling strategy as more of a consecutive sampling than convenient sampling. Please, have a separate subsection for your sampling strategy and explain in detail how this was carried out.

**AUTHORS RESPONSE:** **The authors appreciate your comments. The section has been revised and added to a new subsection study population and subject sampling strategy.**

Have another subsection and define your variables of interest: dependent and independent variables.

**AUTHORS RESPONSE:** **The authors appreciate your comments. The section has been revised**

**Results**

The first row in Table 1 represents means and should be represented as such. The same thing applies to the duration of T2DM. I am not too sure if the remarks on the tables were necessary.

**AUTHORS RESPONSE:** **The authors appreciate your comments. The section has been revised**

**Discussions**

Please, take note that there were several facts stated to which you did not provide references. Example “This relationship has been well substantiated based on the findings that T2DM patients effectively follow diet plans and self-monitor blood sugar levels when they believe in the benefits of undertaking DSM behaviours appropriately”

**AUTHORS RESPONSE:** **The authors appreciate your comments. Reference has been inserted**

Replace "improved for future studies" with "acknowledged"

**AUTHORS RESPONSE:** **The authors appreciate your comments. The section has been revised**

It's not too clear to me why recruiting from a single hospital affects causal relations. This is a serious selection issue "being able to obtain representativeness of the population of interest" I am wondering why the authors expected that by recruiting from a single hospital they will achieve representativeness. population representing the Ghanaian population= are they saying every Ghanaian has the disease.You did not also discuss about information bias. What the authors should note is that selection, information, confounding biases as well as random variation are important determinants of both internal/external validity of their findings and by extension determines the generalizability of the findings. What I was expecting was to discuss how you minimize their presence in your study. I was expecting that the authors will tell readers what they did to minimize the effect of these in their studies. By telling us the problem (not minimizations strategies) in your study you are telling us not to have confidence in your findings. And that will mean this paper is not worth publishing.

**AUTHORS RESPONSE:** **The authors appreciate your comments. The section has been revised based on the reviewers' comments.**

Third, study participants who had difficulties were supported by a qualified research assistant. this is not clear, Check your exclusion criteria

**AUTHORS RESPONSE:** **The authors appreciate your comments. The section has been revised based on the reviewers' comments.**

Despite all the good things you saying about this work if you do not carefully explain selection, confounding, and information biases (random variation) in the context of this work. This paper is worth nothing. Please, do discuss this carefully.

**AUTHORS RESPONSE:** **The authors appreciate your comments. The section has been revised based on the reviewers' comments.**

What is the Global Public Health relevance of your work?

**AUTHORS RESPONSE:** **The authors appreciate your comments. The section has been revised based on the reviewers' comments.**

**Reviewer #2:**

This is a well-written paper and the subject matter is also of importance to the policy makers in ghana considering that NCDs are on the rise. however, the authors need to fix minor issues indicated not Manuscript (see attachment). Few studies in Ghana and the African continent were reviewed and reported in the introduction and discussion sections. The authors could augment the paper with more studies from the sub region.

**AUTHORS RESPONSE:** **The authors appreciate your comments**

Measurement of the model constructs (DSM, level of self-efficacy, belief in treatment efficacy, etc) can be moved to the methods section preferably before the paragraph under "statistical analysis" and the subtitle changed to "Data analysis"

**AUTHORS RESPONSE:** **The authors appreciate your comments. The section has been revised based on the reviewers' comments.**

This sentence can be taken to the introduction section

**AUTHORS RESPONSE:** **The authors appreciate your comments. The section has been revised based on the reviewers' comments.**

Kindly present the findings of your study and the plausible reasons before relating them to others.

**AUTHORS RESPONSE:** **The authors appreciate your comments. The section has been revised based on the reviewers' comments.**

Recommendations can be left for the "Conclusions" section.

**AUTHORS RESPONSE:** **The authors appreciate your comments. The section has been revised based on the reviewers' comments.**

kindly refer to previous comment

**AUTHORS RESPONSE: The authors appreciate your comments. The section has been revised based on the reviewers' comments.**
